# *Staphylococcus aureus* bloodstream infection at a referral children’s hospital in Cape Town, South Africa, 2018–2022

**DOI:** 10.1186/s12879-025-12162-0

**Published:** 2025-11-29

**Authors:** Jombo Namushi, Hafsah Tootla, James Nuttall, Brian Eley

**Affiliations:** 1 Paediatric Infectious Diseases Unit, Red Cross War Memorial Children’s Hospital, Cape Town, South Africa and Department of Paediatrics and Child Health, University of Cape Town, Cape Town, South Africa; 2 Division of Medical Microbiology, National Health Laboratory Service, Red Cross War Memorial Children’s Hospital, Cape Town, South Africa; 3 Division of Medical Microbiology, University of Cape Town, Cape Town, South Africa

**Keywords:** Staphylococcus aureus, Bloodstream infection, Children, Adolescents, Sub-Saharan africa

## Abstract

**Background:**

In the absence of a vaccine, *Staphylococcus aureus (S. aureus)* remains a major cause of morbidity and mortality in children. Studies describing *S. aureus* bloodstream infection (BSI) in children in Africa remain few.

**Methods:**

This was a retrospective descriptive study conducted at Red Cross War Memorial Children’s Hospital (RCWMCH) in Cape Town, South Africa. All *S. aureus* positive blood culture results for the period 2018–2022 on the National Health Laboratory Service microbiology database were identified. Case records of participants with *S. aureus* positive blood cultures were retrieved. Demographic, clinical, antimicrobial management and participant outcome data were extracted from the case records. Continuous variables were presented as means and standard deviations or medians and interquartile ranges as appropriate. Parametric (Student t-test) and non-parametric (Wilcoxon rank sum test for independent variables) methods were used to compare continuous variables. Pearson’s Chi-square or Fisher’s exact tests were used to compare categorical variables. RStudio statistical computing software version 4.4.2 was used for the logistic regression analysis. Our findings were also compared with a similar study at our institution which analysed the epidemiology of Staphylococcus bloodstream infections between 2007 and 2011 (Naidoo et al. PLoS ONE 8(10):e78396, 2013).

**Results:**

During the 5-year study period there were 245 *S. aureus* BSIs identified, with an overall annual incidence risk of 2.7 cases per 1000 admissions. Methicillin-resistant *S. aureus* (MRSA) caused 10% of the BSIs and was predominantly healthcare-associated (79.2%). Only 2.1% of participants were living with human immunodeficiency virus (HIV). However, underweight was present in 29.2% of children with *S. aureus* BSI. Healthcare-associated infection comprised 33.3% of all *S. aureus* BSIs. 70% of BSIs had an identifiable site of infection with skin and soft tissue (25%) being the commonest. All MRSA isolates were susceptible to linezolid, teicoplanin, tigecycline and vancomycin. The mortality rate within 28 days of *S. aureus* BSI diagnosis was 8.3%. The commonest attributable cause of death was septic shock (25%). Requiring intensive care unit (ICU) admission during a BSI, (adjusted Odds ratio (aOR): 5.4, 95% CI: 1.89–15.21) was associated with increased 28-day mortality rate. When compared to the 2007–2011 study, there has been a decline in the incidence risk of *S. aureus* BSI and the prevalence of healthcare-associated MRSA BSI.

**Conclusions:**

*S. aureus* BSI remains a major cause of morbidity and mortality in children. Admission to ICU during a BSI episode was significantly associated with 28-day mortality. Furthermore, over time changes to the epidemiology of *S. aureus* BSI have occurred at RCWMCH.

**Clinical trial number:**

Not applicable (study is not a clinical trial).

**Supplementary Information:**

The online version contains supplementary material available at 10.1186/s12879-025-12162-0.

## Background

Bloodstream infection (BSI) is among the most invasive diseases caused by *Staphylococcus aureus* (*S. aureus*) and is responsible for significant morbidity and mortality in children [1]. Studies describing the epidemiology, clinical manifestations and outcomes of *S. aureus* BSI in Africa have shown variations in disease spectrum and trends over time, even within similar geographical settings, and paediatric studies remain relatively few [2]. In a meta-analysis of bacterial pathogens causing paediatric community-acquired BSI in low- and middle-income countries (LMICs), *S. aureus* was the most common isolate in Africa responsible for 17.8% of BSI [1]. *S. aureus* was the most commonly isolated organism in a 2015 prospective observational study of the aetiology of bacteraemia in young infants < 2 months of age in six countries across 3 continents namely South America (Bolivia), Africa (Ghana, South Africa) and Asia (Bangladesh, India and Pakistan) [3]. Studies from across Africa have identified *S. aureus* as a major cause of BSI in children with isolation rates ranging between 4% and 54% of all cultured isolates [1].

A previous study at Red Cross War Memorial Children’s Hospital (RCWMCH) in Cape Town in 2013 noted that the incidence risk of *S. aureus* BSI was 3.28 cases per 1000 hospital admissions and had remained stable over the five-year study period [4]. *S. aureus* was the leading cause of BSI accounting for 14.9% of all BSIs in children less than 15 years of age in another study at the same institution [5]. In another South African paediatric study, *S. aureus* was the most frequent Gram positive bacterial cause of BSI, accounting for 44% of all Gram positive bacterial isolates [6]. 

Antimicrobial resistance amongst *S. aureus* isolates is a major clinical concern globally [7]. Naidoo et al. at RCWMCH, noted that methicillin-resistant *S. aureus* (MRSA) was responsible for 0.85 cases per 1000 paediatric admissions per year, 26% of all *S. aureus* BSIs, and 72% of MRSA BSIs were nosocomial [4]. The proportion of MRSA infections is increasing worldwide in both children and adult patients [7, 8]. MRSA is mainly a healthcare-associated infection (HA-MRSA), but community-acquired MRSA (CA-MRSA) infection is increasing in frequency [9, 10]. In South Africa, studies in children and laboratory surveillance in children and adults have reported an increasing prevalence of MRSA infection from 2%, to 31% to 59% in 1974, 1995, and 2010 respectively [11, 12]. Musicha et al. in Malawi noted a more than two-fold increase in MRSA BSI from 7.7% in 1998 to 18.4% in 2016 [13]. MRSA isolates in children with BSI are often multidrug resistant [4, 10]. 

In this study, we describe the recent epidemiology, clinical spectrum, treatment, drug susceptibility patterns and outcome of *S. aureus* BSI among children at RCWMCH between January 2018 and December 2022, as well as recent changes to the profile of *S. aureus* BSI at the hospital when compared with findings of a previous study of children with *S. aureus* BSI diagnosed between January 2007 and December 2011 at RCWMCH [4]. 

## Methods

### Study setting

The study was conducted at RCWMCH, a 292-bed tertiary level referral hospital primarily serving sick children from the Western Cape and surrounding provinces. It caters for children below the age of 13 years, providing both general paediatric services and highly specialised care including infectious diseases services.

### Study design

This retrospective study reviewed case records and laboratory results of participants admitted between January 2018 and December 2022 with blood culture-confirmed *S. aureus* BSI.

### Data collection

The academic and research unit managing the Central Data Warehouse (CDW) in the information technology department of the National Health Laboratory Service (NHLS) in Johannesburg, South Africa retrieved the list of children admitted to RCWMCH with laboratory-confirmed *S. aureus* BSI during the study period. This list was used to obtain the clinical records and laboratory results (including HIV test results, blood culture confirmation, antimicrobial susceptibility results, full blood count and C - Reactive protein results at the time of diagnosis) from every child with *S. aureus* BSI during the study period.

Paper-based medical records of children with *S. aureus* BSI at RCWMCH were reviewed and relevant data were extracted including demographic information, clinical information at presentation and during the course of *S. aureus* BSI, prior antibiotic exposure, factors predisposing to BSI, the type of BSI (infection present on admission or hospital-acquired infection), the site of infection, comorbidities, complications, antibiotic treatment, outcome, and thereby factors associated with outcome were determined. All data were entered onto standardised, study-specific data collection sheets. Recurrent episodes of S. aureus BSI were reported as separate episodes.

### Study definitions

Definitions used in this study are described and referenced in Supplementary Table 1.

### Microbiological procedures

Microbiology testing was conducted at the NHLS clinical microbiology laboratory at Groote Schuur Hospital. For the study duration (2018–2022), the BACT/ALERT PF Plus and FA Plus blood culture bottles were used where appropriate along with the BACT/ALERT automated blood culture system (bioMerieux Inc., Durham, NC, USA). Positive blood cultures were Gram-stained and sub-cultured onto appropriate standardized media. Growth of organisms were either identified using the automated Vitek^®^2 identification system (bioMérieux, Inc., France), or standardized biochemical methods (Pastorex™ Staph-Plus (BIO-RAD) and DNAse production). Catalase was also used to confirm that the gram positive cocci (GPC) were *Staphylococcus species*. Antibiotic susceptibility testing (AST) was performed with the automated Vitek^®^2 AST system (bioMérieux, Inc., France). The Vitek^®^2 AST-P603 card was used and tests the following anti-staphylococcal antibiotics: cloxacillin, penicillin/ampicillin, erythromycin/azithromycin, fusidic acid, gentamicin, ciprofloxacin, clindamycin, linezolid, moxifloxacin, rifampicin, teicoplanin, tetracycline, tigecycline, trimethoprim-sulfamethoxazole and vancomycin. Occasionally, were appropriate disk diffusion or vancomycin gradient diffusion tests were performed to confirm discrepant AST results from the automated Vitek^®^2 AST system (bioMérieux, Inc., France). Antimicrobial susceptibility test results were interpreted using interpretative criteria published by the Clinical and Laboratory Standards Institute for the relevant year and breakpoints published by the Food and Drug Administration for tigecycline [14]. 

### Data processing and analysis

Data was transferred from the data collection sheets to a dedicated excel file and analysed in Stata/BE version 18.0 for Windows (College Stata, TX, USA). Continuous variables were presented as means and standard deviations or medians and interquartile ranges as appropriate. Categorical variables were summarised using proportions and percentages. Parametric (Student t-test) and non-parametric (Wilcoxon rank sum test for independent variables) methods were used to compare continuous variables. Pearson’s Chi-square or Fisher’s exact tests were used to compare categorical variables. Incidence risk was calculated per 1000 hospital admissions. The RCWMCH Management Information Unit provided the number of hospital admissions for each year of the study period. These admission numbers were used in the incidence risk calculations. Factors associated with outcome were evaluated using univariable and multivariable logistic regression. P-value and 95% confidence interval were used to determine statistical significance. RStudio statistical computing software version 4.4.2 (2024-10-31 ucrt, copyright © 2024 The R Foundation for Statistical Computing Platform: x86_64-w64-mingw32/x64) was used for logistic regression. Variables/covariates were preselected by the investigator based on published research findings. The selected variables were age, sex, comorbidities, healthcare-associated BSI (HA-BSI), infection involving 2 or more systems, initiation of effective antibiotic therapy within 24 h, ICU admission during BSI, under-weight (defined as weight-for-age Z-score (WFAZ) < -2), haemoglobin < 7 g/dl, and HIV status (unknown/known). All the variables were made binary for the univariable analysis. The same variables were used in the multivariable logistic regression analysis.

The findings of this study were compared with a similar retrospective study at our institution which analysed the epidemiology of *Staphylococcus aureus* bloodstream infections between 2007 and 2011 [4]. 

## Results

### Study participants

**Fig. 1:**
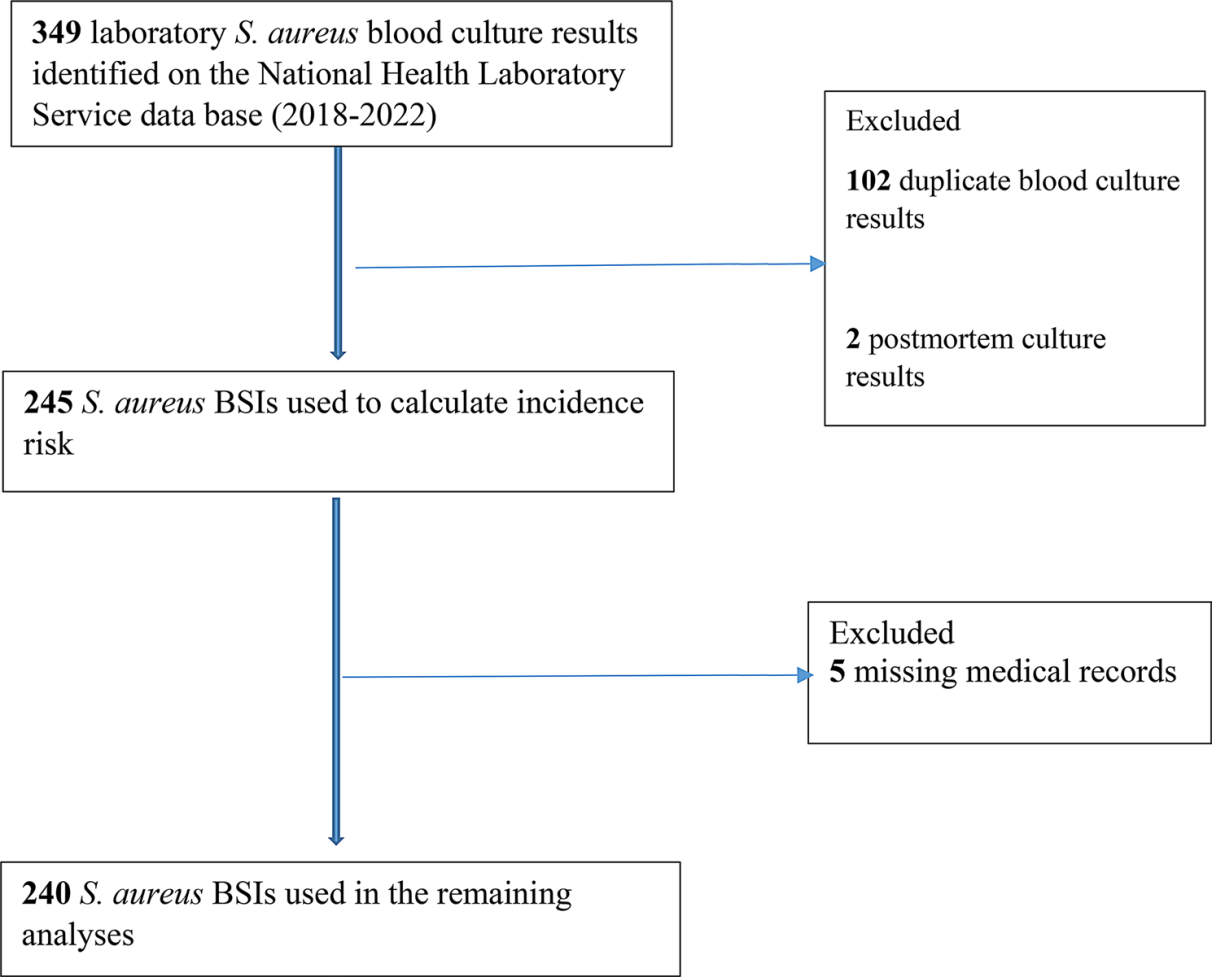
Selection of *Staphylococcus aureus* bloodstream infection episodes for the data analysis

There were 349 *S. aureus* positive blood culture laboratory results for RCWMCH during the 5-year study period in the NHLS CDW. After duplicate and postmortem culture results were excluded, there was a total of 245 *S. aureus* BSI episodes during the study period, which was used to calculate the incidence risk. Five medical records were missing. Therefore, clinical and laboratory data from the remaining 240 *S. aureus* BSI episodes were used in the remaining analyses, 2 of which were recurrent episodes (Fig. 1).

### Incidence risk of *S. aureus* bloodstream infections

**Fig. 2:**
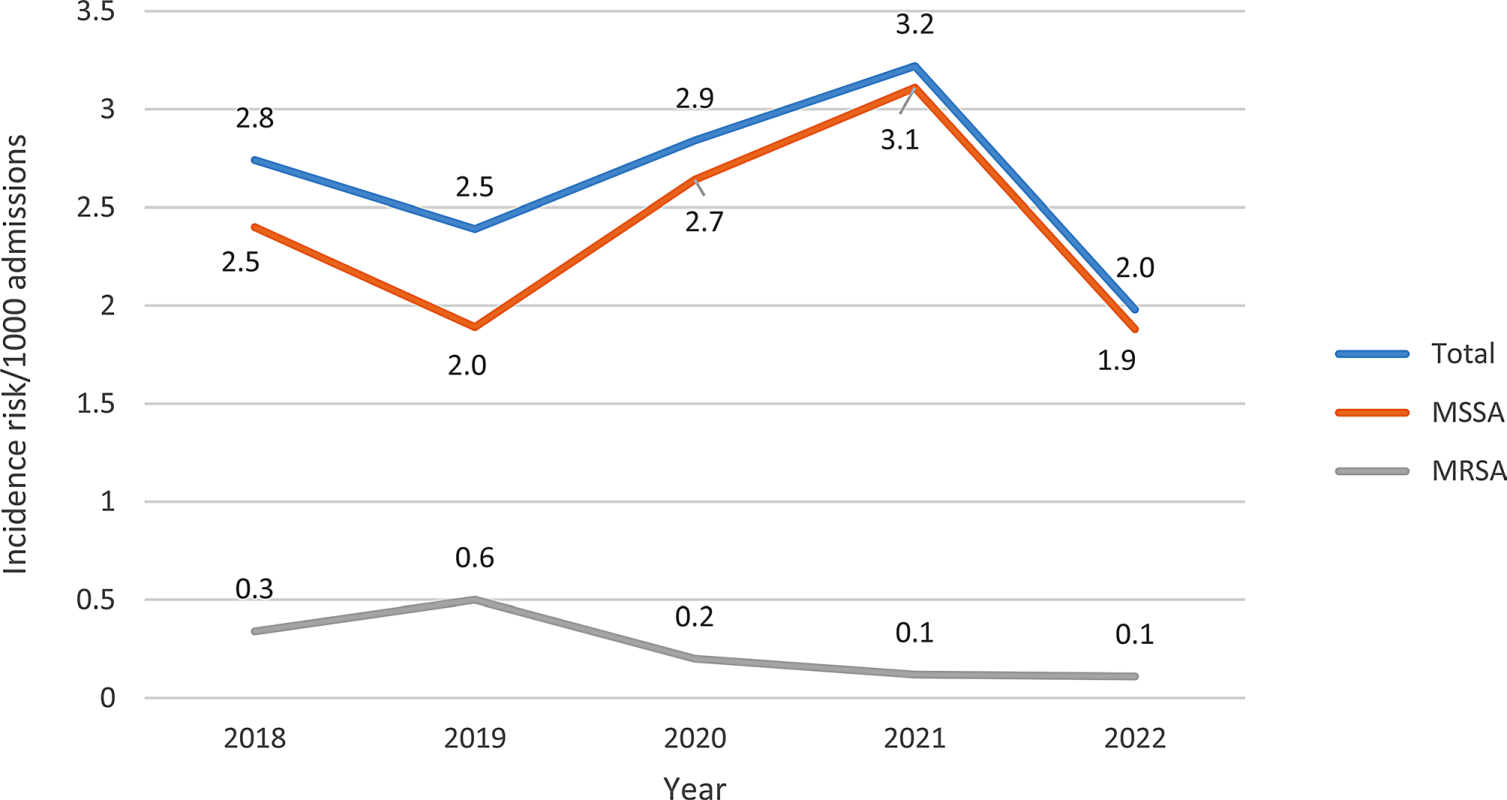
*Staphylococcus aureus* bloodstream infection incidence risk/1000 admissions January 2018 – December 2022. MSSA, methicillin-susceptible *Staphylococcus aureus*; MRSA, methicillin-resistant *Staphylococcus aureus*

Over the 5-year study period, there was an overall incidence risk of 2.7 *S. aureus* BSIs per 1000 hospital admissions. The incidence risk was highest in 2021 (3.2) and lowest in 2022 (2.0). Methicillin-susceptible *S. aureus* (MSSA) was the cause of 89.8% (220) of all BSIs, while 10.2% (25) were caused by MRSA isolates (Fig. 2 and Supplementary Table 2).

### Baseline characteristics of study participants

Table 1 describes the characteristics of the 240 *S. aureus* BSIs with available medical records. The median age at diagnosis was 15.5 (IQR 3.3–79.3) months. Participants with MRSA BSI were significantly younger (median 5.1 (1.7–14.1) months) compared to those with MSSA BSI (median 17.7 (3.9–83.0) months, *p* = 0.01). Infants (children < 12 months of age) were the predominant participant group (45%), of which 20% (22/108) were neonates. Underweight-for-age was present in 29.2% of the participants.

**Table 1:** Characteristics of patients at the time of *Staphylococcus aureus* bloodstream infection

Characteristic	All S. aureus BSIs (*N* = 240 unless stated)	MSSA BSIs (*N* = 216 unless stated)	MRSA BSIs (*N* = 24 unless stated)	*p* value*
Median age in months (IQR)	15.5 (3.3–79.3)	17.7 (3.9–83.0)	5.1 (1.7–14.1)	0.01
Age category, number (%)				0.03
<1 month	22 (9.2)	20 (9.3)	2 (8.3)	
*≥*1 month-1 year	86 (35.8)	71 (32.9)	15 (62.5)	
*≥*1–5 years	66 (27.5)	61 (28.2)	5 (20.8)	
>5 years	66 (27.5)	64 (29.6)	2 (8.3)	
Male: Female, number (%)	146 (60.8): 94(39.2)	135 (62.5): 81 (37.5)	13 (54): 11 (46)	0.4
Median weight-for-age Z –score (IQR)	-1.1 (-2.2–0.08)	-1.0 (-2.1–0.1)	1.8 (-2.8 - -0.2)	0.1
Under weight-for-age <-2 Z score (%)	70 (29.2)	60 (27.8)	10 (41.7)	0.2
HIV status, number (%)				0.08
HIV infected	5 (2.1)	5 (2.3)	0 (0)	
HIV exposed uninfected	50 (20.8)	40 (18.5)	10 (41.7)	
HIV unexposed and uninfected	142 (59.2)	130 (60.2)	12 (50)	
HIV status unknown	43 (17.9)	41 (19)	2 (8.3)	
Antibiotic exposure in the 12-month period before onset of BSI, number (%)	39 (16.3)	33 (15.3)	6 (25)	0.2
Temperature category at BSI diagnosis, number (%)	*N* = 234	*N* = 210	*N* = 24	
Temperature > 37.5 °C	148 (63.2)	132 (62.9)	16 (66.7)	0.9
Temperature 35.0–37.4 °C	84 (35.9)	76 (32.2)	8 (33.3)	
Temperature < 35 °C	2 (0.9)	2 (1.0)	0 (0)	
Median white cell count x 10^9^/L (IQR)	*N* = 235 13.8 (9.0–20.6)	*N* = 211 14.3 (8.7–20.5)	*N* = 24 13.7 (11.4–22.3)	0.4
White cell count > 20 × 10^9^/L (%)	64 (27.2)	57 (27.0)	7 (29.2)	0.8
Median neutrophil count x 10^9^/L (IQR)	*N* = 167 6.9 (3.5–12.1)	*N* = 150 6.8 (3.0–11.9)	*N* = 17 9.1 (8.0–14.9)	0.07
Neutrophil count < 2.0 × 10^9^/L, number (%)	21 (12.6)	20 (9.5)	1 (5.9%)	0.7
Median haemoglobin in g/dL (IQR)	*N* = 235 10.2 (9.1–11.9)	*N* = 211 10.4 (9.1–12.0)	*N* = 24 9.9 (8.2–10.5)	0.1
Haemoglobin category, number (%)	*N* = 235	*N* = 211	*N* = 24	0.1
Haemoglobin ≤7 g/dL (severe anaemia)	12 (5.1)	11 (5.2)	1 (4.2)	
Haemoglobin 7–11 g/dL (mild anaemia)	131 (55.7)	113 (53.6)	18 (75)	
Haemoglobin ≥11 g/dL (no anaemia)	92 (39.1)	87 (41.2)	5 (20.8)	
Median CRP in mg/L (IQR)	*N* = 177 147 (40–244)	*N* = 166 154 (44–252)	*N* = 11 45 (15–161)	0.05
CRP > 10 mg/L, number (%)	156 (88.1)	147 (88.6)	9 (81.8)	0.6

BSIs, bloodstream infections; *S. aureus*,* Staphylococcus aureus*; MSSA – Methicillin-susceptible *Staphylococcus aureus*, MRSA – Methicillin-resistant *Staphylococcus aureus*; IQR, interquartile range; HIV, Human Immunodeficiency virus; CRP, C-reactive protein; * comparison of MSSA and MRSA BSIs

Table 1. Characteristics of study participants at the time of *Staphylococcus aureus* bloodstream infection.

### Classification and clinical spectrum of *S. aureus* bloodstream infections

Predictably, most community-acquired *S. aureus* BSIs were caused by MSSA isolates, while most healthcare-associated BSIs were caused by MRSA isolates as shown in Table 2. Healthcare-associated BSIs accounted for 33.3% of the total number of *S. aureus* BSIs. Furthermore, a higher percentage of BSIs caused by MSSA isolates had an identifiable focus of infection compared to those BSIs caused by MRSA isolates, 155/216 (71.8%) vs. 12/24 (50%), *p* = 0.03.

**Table 2:** Classification and clinical spectrum of *Staphylococcus aureus* bloodstream infections

Clinical diagnosis (%)	All S. aureus BSIs Number (%) (*N* = 240)	MSSA BSIs Number (%) (*N* = 216)	MRSA BSIs Number (%) (*N* = 24)	*P* value*
Community-acquired BSI	160 (66.7)	155 (71.8)	5 (20.8)	< 0.001
Healthcare-associated BSI	80 (33.3)	61 (28.2)	19 (79.2)	
BSI without identifiable focus of infection	73 (30.4)	61 (28.2)	12 (50)	0.03
BSI with focus of infection	167 (69.6)	155 (71.8)	12 (50)	
Primary diagnosis/site of infection				
No focus identified	73 (30.4)	61 (28.2)	12 (50)	0.05
Skin and soft tissue infection	60 (25)	52 (11.6)	8 (33.3)	
Pneumonia	24 (10)	23 (10.6)	1 (4.2)	
Pericarditis/endocarditis	4 (1.7)	3 (1.4)	1 (4.2)	
Septic arthritis	19 (7.9)	19 (8.8)	0 (0)	
Osteomyelitis	14 (5.8)	14 (6.5)	0 (0)	
Gastroenteritis	21 (8.8)	21 (9.7)	0 (0)	
Meningitis	3 (1.3)	2 (0.9)	1 (4.2)	
Infections involving 2 or more systems	22 (9.2)	21 (9.7)	1 (4.2)	

*S. aureus*,* Staphylococcus aureus*; MSSA, methicillin-susceptible *Staphylococcus aureus*; MRSA, methicillin-resistant *Staphylococcus aureus*; BSIs, bloodstream infections; * comparison of MSSA and MRSA BSIs

### Antimicrobial susceptibility profile of MSSA and MRSA isolates

216/240 (90%) of the *S. aureus* isolates were susceptible to cloxacillin (MSSA isolates); the remaining 24 (10%) isolates were resistant to cloxacillin (MRSA isolates). Of the 216 MSSA isolates 84.3% (182/216) were resistant to penicillin/ampicillin. Antibiotic resistance was variable among isolates, but all tested isolates were susceptible to linezolid, teicoplanin, tigecycline, and vancomycin (Table 3).

**Table 3:** Antimicrobial susceptibility profile of MSSA and MRSA isolates

Antibiotic tested	MSSA isolates		MRSA isolates	
	Number (%) of resistant isolates	Number of isolates tested	Number (%) of resistant isolates	Number of isolates tested
Ciprofloxacin	0 (0)	216	12 (54.5)	22
Clindamycin	12 (5.6)	216	13 (59.1)	22
Erythromycin/Azithromycin	15 (6.9)	216	13 (59.1)	22
Fusidic acid	3 (1.4)	216	1 (4.5)	22
Gentamicin	5 (2.3)	216	12 (54.5)	22
Linezolid	0 (0)	216	0 (0)	22
Moxifloxacin	0 (0)	216	9 (40.9)	22
Penicillin/Ampicillin	182 (84.3)*	216	23 (100)	23
Rifampicin	1 (0.5)	216	2 (8.7)	23
Teicoplanin	0 (0)	216	0 (0)	22
Tetracycline	11 (5.1)	216	9 (40.9)	22
Tigecycline	0 (0)	216	0 (0)	22
Trimethoprim/sulfamethoxazole	89 (41.2)	216	8 (36.4)	22
Vancomycin	0 (0)	216	0 (0)	24

MSSA, methicillin-susceptible *Staphylococcus aureus*; MRSA, methicillin-resistant *Staphylococcus aureus*

*This value may be under reported as penicillin sensitivity of all VITEK2 reported penicillin sensitive isolates are not routinely confirmed with penicillinase testing in our setting. Isolates that are reported as penicillin sensitive by the VITEK2 are not reported to the clinician and penicillin is not used as a treatment option for S. aureus infection

### Antimicrobial therapy

Information on antibiotic therapy was available for all 240 episodes. Effective antibiotic therapy was prescribed in 226 (94.2%) of the BSIs. In the remaining 14 BSIs, effective therapy was not administered, either empirically or definitively at our facility and all the 14 comprised of MSSA episodes. In seven of these 14 BSIs, the study participants died before blood culture results were known, 6 participants died on arrival, while 1 died within 24 h of admission. Of the remaining 7 who did not receive any effective antibiotic therapy at our facility, 3 were transferred to other facilities to complete antibiotics before blood culture results were available, another 3 were discharged before blood culture results were known, while in 1 participant the *S. aureus* isolate was considered a contaminant and was not treated. Instead, the participant was treated for complications of cerebral palsy and discharged after 7 days. Of the participants who received effective therapy, 74% (168/226) initiated effective therapy on day zero (day blood culture was taken), 3% (5/168) of whom had MRSA, while 97% (163/168) had MSSA. The median time between blood culture collection and initiation of effective therapy for both MSSA and MRSA groups was within 24 h (median 0 (0–1) days) of BSI diagnosis. The median duration of effective therapy was 14 (9.5–24.5) days and was shorter for BSI without a focus of infection, median 14 (5.5–14) days compared to BSI with an identifiable focus, median 17 (11.5–32) days, *p* < 0.001.

The most frequently administered empiric antimicrobial therapy for MSSA isolates was a third-generation cephalosporin, either ceftriaxone or cefotaxime (26%), followed by cloxacillin (12%) or a combination of piperacillin-tazobactam and amikacin (12%). 19% (41/216) of the participants with MSSA BSI did not initiate empiric therapy and were initiated on definitive therapy once the blood culture result was available, this category mainly comprised of patients with hospital associated BSI being managed for another condition such as malignancy, cardiac condition, or burns. The median time to initiation of definitive antibiotic therapy was 0 (0–0.5) days as definitive therapy was initiated within 24 h of BSI diagnosis. Most preliminary blood culture results were notified by the microbiology laboratory within 24 h of collection enabling initiation of definitive therapy. Regardless of empiric antibiotic therapy, in 89% (156/175) of MSSA episodes, antibiotics were de-escalated to cloxacillin for directed antibiotic therapy (Table 4).

46% of MRSA episodes (11/24) were not initiated on empiric antibiotic therapy. However, definitive therapy with vancomycin was initiated in all cases once the blood culture results were available (median time to definitive therapy was 0 (0–1) days – initiated within 24 h of BSI diagnosis). For participants who initiated empiric therapy, a combination of piperacillin-tazobactam and amikacin was the commonest empiric therapy, administered in 29% (7/24) of MRSA episodes, followed by a third-generation cephalosporin (12.5%) and vancomycin (8.3%). In all MRSA episodes, antibiotics were changed to vancomycin for definitive therapy if they were not already on vancomycin empirically.

**Table 4:** Antimicrobial therapy participants received during the *S. aureus* BSI episode

Empiric antibiotic therapy				Definitive antibiotic therapy			
MSSA BSIs, number (%) (*N* = 216)		**MRSA BSIs**,** number (**%**)(*N*** **=** **24)**		**MSSA BSIs**,** number (**%**) (*N*** **= 216)**		**MRSA BSIs**,** number (**%**) (*N*** **= 24)**	
3rd generation cephalosporin*	57 (26)	None	11 (46)	Cloxacillin	156 (72)	Vancomycin	24 (100)
None	41 (19)	Piperacillin- tazobactam & amikacin	7 (29)	None	14 (6.5)		
Cloxacillin	26 (12)	3rd generation cephalosporin	3 (12.5)	Vancomycin	10 (4.6)		
Piperacillin- tazobactam & amikacin	26 (12)	Vancomycin	2 (8.3)	1st generation cephalosporin	8 (3.7)		
Ampicillin & gentamicin	21 (9.7)	Carbapenem	1 (4.2)	3rd generation cephalosporin	8 (3.7)		
Carbapenem	16 (7.4)			Flucloxacillin	7 (3.2)		
Co-amoxiclav	12 (5.6)			Co-amoxiclav	7 (3.2)		
Vancomycin	9 (4.2)			Piperacillin- tazobactam & amikacin	3 (1.4)		
1st generation* cephalosporin	6 (2.8)			Carbapenem	3 (1.4)		
Amoxicillin	2 (0.1)						

BSIs – bloodstream infections, ^*^1st generation cephalosporin – cefazolin and cephalexin, ^*^3rd generation cephalosporin – ceftriaxone and cefotaxime, Carbapenem – meropenem and ertapenem. MSSA – methicillin-susceptible *Staphylococcus aureus*, MRSA – methicillin-resistant *Staphylococcus aureus*, co-amoxiclav – amoxicillin/clavulanic acid

### Outcomes

Table 5 summarises the outcome of the 240 *S. aureus* BSIs. The mortality rate within 28 days of *S. aureus* BSI diagnosis was 8.3%. (20/240). All deaths within 28 days occurred in participants with MSSA BSI. Six participants died on arrival at the hospital, another 2 died within 24 h of admission. Sepsis/septic shock was the commonest attributable cause of death and was identified in 55% (11/20) of cases. 40% (8/20) of participants that died had healthcare-associated *S. aureus* BSI. Furthermore, 65% (13/20) of participants who died had co-morbidities (supplementary Table 3), notably congenital cardiac disease in 23% (3/13). The median WFAZ for participants who died was − 1.13 (-2.27–0.07), with 35% (7/20) being moderately or severely underweight.

**Table 5:** Outcomes of participants with *Staphylococcus aureus* bloodstream infection

Outcome of patient with BSI	All S. aureus BSIs, number (%) (*N* = 240)	MSSA BSIs, number (%) (*N* = 216)	MRSA BSIs, number (%) (*N* = 24)	*P*
Overall outcome				0.8
In-patient mortality	25 (10.4)	23 (10.6)	2 (8.3)	
Discharge from hospital	187 (77.9)	167 (77.3)	20 (83.3)	
Transfer out to complete antibiotics	28 (11.7)	26 (12.0)	2 (8.3)	
28-day mortality	20 (8.3)	20 (9.3)	0	0.2

*S. aureus*,* Staphylococcus aureus*; MSSA, methicillin-susceptible *Staphylococcus aureus*; MRSA, methicillin-resistant *Staphylococcus aureus;* BSIs, bloodstream infections

45% (9/20) of the deaths occurred in participants with BSI without a focus, while pneumonia occurred in 44% (4/11) of the participants who died with an identifiable focus of infection. None of the participants who died were living with HIV, while 35% (7/20) had unknown HIV status. Effective antibiotic therapy was initiated on day zero (same day blood culture was taken) in 60% (12/20) of participants who died. Of the remaining 8 participants, six did not receive antimicrobial therapy because they died shortly after arriving at the hospital, while the remaining 2 initiated therapy to which the *S. aureus* isolate was not susceptible.

### Risk factors for mortality

Table 6 describes risk factors for 28-day in-patient mortality among participants with *S. aureus* BSI. On univariable analysis, the presence of comorbidities (Supplementary Table 3), admission to ICU during a BSI episode and being underweight (WFAZ < -2) were significantly associated with 28-day mortality. Furthermore, the presence of BSI with a focus reduced the 28-day mortality risk. On multivariable analysis, admission to ICU during a BSI episode remained statistically significant and unknown HIV status became significantly associated with 28-day mortality.

**Table 6:** Risk factors for mortality

Risk factors for mortality	Univariable analysis		Multivariable analysis	
	Unadjusted Odds ratio	95% CI	Adjusted Odds ratio	95% CI
Sex: Female : Male	Ref 0.8	0.35–1.88	Ref 1.07	0.63–3.81
Age: *≤*12 months	1.9	0.97–3.01	1.7	0.99–1.01
: >12 months	Ref		1	
Comorbidities*	3.5	1.46–9.34	3.56	0.67–9.85
HA-BSI	1.66	0.7–3.84	0.49	0.29–2.96
Infection involving two or more systems	0.8	0.12–2.99	0.9	0.23–5.46
Disease with identifiable focus	0.33	0.14–0.76	0.72	0.32–1.11
Effective antibiotics within 24 h (day zero)	0.45	0.18–1.9	0.7	0.41–1.56
ICU admission	3.14	1.31–7.4	5.4	1.89–15.21
Unknown HIV status	2.35	0.9–5.74	5.06	1.95–11.51
Underweight (WFAZ <-2)	2.5	1.07–5.82	2.17	0.96–4.31
Haemoglobin < 7 g/dL	4.44	0.2–48.01	3.42	0.41–68.21

HA, healthcare-associated; BSI, bloodstream infection; ICU, intensive care unit; HIV, human immunodeficiency virus; WFAZ, weight-for-age Z-score; CI, confidence interval; comorbidities*, refer to supplementary Table 3

### Comparison of *S. aureus* bloodstream infection during the periods 2007–2011 and 2018–2022

The epidemiology of *S. aureus* BSI at RCWMCH has changed over time. The annual incidence risk has declined from 3.3/1000 admissions in the 2007–2011 study period to 2.7/1000 admissions in the current study period. There has been a significant decline in the HIV infection rate, the proportion of *S. aureus* BSIs with unknown HIV status and the percentage of BSIs caused by MRSA. On the contrary, the 28-day mortality remains similar at 8.3% in the current study compared to 8.8% in the previous study (Supplementary Table 4).

## Discussion

In this retrospective study we described blood-culture confirmed *S. aureus* BSI in children admitted to RCWMCH between January 2018 and December 2022. The findings show that *S. aureus* BSI remains an important cause of morbidity and mortality in children and that the epidemiology of *S. aureus* BSI at our hospital has changed over time.

The incidence of *S. aureus* BSI was 2.7/1000 admissions (supplementary Table 2). A previous study at our hospital recorded a higher incidence of 3.3/1000 admissions demonstrating a 20% reduction in the incidence risk between the two study periods [4]. In our study, MRSA comprised 10.2% of *S. aureus* BSI demonstrating a 60% reduction from the previous of study (26%) [4]. This is in contrast to global trends of increasing antimicrobial resistant pathogens including MRSA [7]. The recent decline in the incidence risk for *S. aureus* BSI at our hospital may be partly attributed to the impact of the COVID-19 pandemic as the study period includes the COVID-19 pandemic years. During the COVID-19 pandemic there were restricted hospital visits and heightened infection prevention measures at all levels including healthcare facilities. This might have led to an overall reduction in cases, particularly healthcare-associated MRSA infections. General improvement in infection control practice and antimicrobial stewardship at RCWMCH, likely contributed to the lower incidence risk.

45% of the participants were infants. This is similar to findings from other South African studies demonstrating younger age as a risk factor for *S. aureus* BSI especially neonates being at an increased risk of MRSA BSI [15–17]. In some high-income countries, the average age of children with *S. aureus* BSI is relatively higher than in our study. For example, studies from Spain and Australia documented a mean age of 5.6 years and median age of 57 months respectively compared to a median age of 15.5 months in our study [18, 19]. The previous study at our hospital reported a median age of 11.3 months among participants with *S. aureus* BSI. Another study from South Africa reported a median age of 7.5 months and in a study from the Gambia, 73% of participants with *S. aureus* bacteraemia were infants suggesting that the younger age group may be more at risk for *S. aureus* BSI in low- and middle-income countries compared to high-income countries [4, 10, 20]. 

In our study, the HIV infection prevalence of 2.1% is substantially lower than that reported in the 2007–2011 study. A likely explanation for this decrease in HIV infection prevalence is the impact of the South African prevention of mother-to-child transmission (PMTCT) programme in reducing vertical transmission from women living with HIV to their offspring [21]. 

As evident in our study, the clinical presentation of *S. aureus* BSI may vary. 30% of the BSIs had no clinical focus of infection, and of those with a focus, skin and soft tissue infections or pneumonia were common. These findings are similar to the previous study in which 33% of *S. aureus* BSI had no identifiable focus, while pneumonia and empyema were the most common identifiable foci of infection [4] Another study among children in South Africa reported similar findings, with pneumonia being the most common identifiable focus of infection [10]. However, a study from Australia and New Zealand found that bone and joint infection was the most common primary clinical manifestation (34%), with sepsis without a focus in second place (21%) [18].

20% of MRSA isolates in our study were community-acquired, and most MRSA isolates were resistant to multiple antibiotics. All isolates were susceptible to vancomycin, linezolid, teicoplanin and tigecycline. These findings are similar to the previous study at the same institution [4]. A study in Spain also demonstrated variable resistance to most antibiotics tested, but all isolates were susceptible to rifampicin, teicoplanin and vancomycin [19]. In the 2019 global burden of bacterial antimicrobial resistance report, *S. aureus* was cited among the top six leading pathogens causing antimicrobial resistance-associated mortality [7]. The low susceptibility rates of other commonly used drugs in the management of *S. aureus* infections such as clindamycin and trimethoprim-sulfamethoxazole limit their empiric use, while the high susceptibility of vancomycin substantiates its role as the drug of choice for MRSA infection in children in our setting.

Effective antibiotic therapy was initiated on day zero (within 24 h) of diagnosis of the BSI in 74% of participants who received effective antibiotic therapy and was similar for MSSA and MRSA episodes (median 0 days, IQR 0–1 day – within 24 h of diagnosis). This finding contrasts with the previous study at the same institution which reported a shorter duration to effective therapy for MSSA episodes (median time 2.5 h) compared to MRSA episodes (median time 46 h) [4]. Our study did not find any difference in time to effective therapy between MSSA and MRSA episodes because we used days as the time unit, hours would have been preferred and better for comparison with previous findings. Most MRSA episodes were healthcare-associated (79.2%) hence the most common empiric therapy was piperacillin-tazobactam and amikacin, in accordance with the hospital antibiotic policy for healthcare-associated infections. The most common antibiotic prescribed for MSSA episodes was a third-generation cephalosporin. This might have been influenced by the age of the participants, 45% being infants, 20% of whom were neonates. Third- generation cephalosporins (cefotaxime for neonates and ceftriaxone for infants) are the drugs of choice for sepsis when meningitis is not excluded in young children [22]. This contrast with the previous study which reported penicillin and gentamicin as the most prescribed empiric therapy [4]. 46% of MRSA BSIs were not initiated on empiric antibiotics; however effective therapy was initiated within 24 h upon receipt of blood culture results. This was made possible through a strong collaboration between the laboratory and the infectious diseases clinicians which exists at the institution enabling timely communication of laboratory results and prompt initiation of appropriate therapy in line with laboratory results. By comparison, a study in Australia and New Zealand reported flucloxacillin, a penicillinase-resistant penicillin as the most frequently prescribed antibiotic for *S. aureus* BSI in 77% of cases, [18] while in Spain, another high-income country, vancomycin was the most commonly used empiric treatment in 31% of the cases with an MRSA prevalence ranging from 3% to 14% over the 18 years study period [19]. A survey from Australia and New Zealand reported variations in the choice of antibiotics for the treatment of *S. aureus* bacteraemia among infectious diseases clinicians [23]. Furthermore, the management of *S. aureus* bacteraemia in children is mainly based on clinical experience and extrapolation of evidence from adult trials, as there is limited high-quality evidence in children to guide management [24]. 

The 28-day mortality documented in our study is comparable to that documented in the previous study at our institution, refer supplementary Table 4 [4]. Unknown HIV status and admission to ICU were significant risk factors for 28-day mortality. Admission to the ICU implies severe illness requiring invasive monitoring and/or interventions such as ventilatory support for respiratory failure or inotropes for septic shock, which were frequent complications in participants who died. Unknown HIV status was significantly associated with mortality on multivariable analysis, this could be explained by the fact that 40% of deaths occurred on day zero (within 24 h of admission) or upon arrival at the hospital before participants were tested for HIV infection. We therefore believe that the true causal relationship was not that unknown HIV status increased the risk of dying, rather that dying early increased the chance of not having an HIV test done, as a good proportion of participants died early before establishing their HIV status. In the previous study at our institution, only MRSA infection was significantly associated with mortality [4]. In our study, all participants who died within 28 days of BSI diagnosis had MSSA infection. Of note we had two participants with recurrent BSIs making it 2 episodes and are accounted for in the total number of episodes. The number however was too small for further analysis.

Our study is not without limitations, however, a strength of this study is that we were able to compare our results with those of a previous study conducted at our hospital and describe differences in the epidemiology of *S. aureus* BSI between the two study periods. Due to the retrospective nature of the study, there were limitations in the completeness and availability of clinical information and laboratory results for some of the BSIs. Furthermore, the practice of administering broad-spectrum third-generation cephalosporins to sick children at primary and secondary healthcare facilities before transfer to our hospital, implies that the true burden of community-acquired *S. aureus* BSI may have been underestimated. Our time unit for duration to effective therapy was in days, this might have contributed to the non-significance of this variable in various analyses, hours would have been a preferred time unit. Additionally, because of low 28-day mortality, the risk factor analysis was underpowered to identify all risk factors associated with 28-day mortality. Despite these limitations, the study findings do provide valuable information on *S. aureus* BSI at our hospital.

## Conclusion

The study demonstrated that *S. aureus* BSI remains an important cause of morbidity and mortality among children at our institution and that the epidemiology has changed over time. In a world of rising antimicrobial resistance, the marked reduction in MRSA prevalence reported at our institution is commendable. Even though the findings may not be generalized to all referral hospitals in LMICs, they provide important insights. Future prospective studies are warranted to better understand the epidemiology of *S. aureus* infections in children, including risk factors associated with mortality. Inclusion of children in *S. aureus* BSI treatment clinical trials is needed to optimise antimicrobial therapy.

## Supplementary Information

Below is the link to the electronic supplementary material.

Supplementary Material 1

## Data Availability

The data that support the findings of this study are available from RCWMCH and the NHLS, South Africa, but restrictions apply to the availability of these data, which were used under permission for the current study, and so are not publicly available. Data are however available from the authors upon reasonable request and with permission of RCWMCH and NHLS.

## Abbreviations

ART: Antiretroviral therapy
AST: Antimicrobial susceptibility testing
BSI: Bloodstream infection
CA: MRSA-Community-acquired methicillin-resistant Staphylococcus aureus
CFR: Case fatality rate
CLSI: Clinical and Laboratory Standards Institute
CRP: C-reactive protein
HA: MRSA-Healthcare-associated methicillin-resistant Staphylococcus aureus
HIV: Human Immunodeficiency Virus
HREC: Human research ethics committee
IQR: Interquartile range
LMICs: Low-and middle-income countries
MRSA: Methicillin-resistant Staphylococcus aureus
MTCT: Mother-to-child transmission
NHLS: National Health Laboratory Service
PMTCT: Prevention of mother-to-child transmission
RCWMCH: Red Cross War Memorial Children’s Hospital
S. aureus: Staphylococcus aureus
WFAZ: Weight-for-age Z-score
