# Supplementary material for: Staphylococcus aureus bloodstream infection at a referral children’s hospital in Cape Town, South Africa, 2018–2022
Source: BMC Infect Dis. 2025 Nov 29;26:5. doi: 10.1186/s12879-025-12162-0 (PMC12771707; doi:10.1186/s12879-025-12162-0)
Supplement: Supplementary file 1 — Supplementary Material 1 [file 12879_2025_12162_MOESM1_ESM.docx]

**Supplementary file**

**Supplementary table 1.** Study definitions

| 1. Anaemia: blood haemoglobin concentration <11 g/dL.^1^ |
| --- |
| 2. Anti-staphylococcal antibiotics include oxacillin, cloxacillin, piperacillin/tazobactam, amoxicillin/clavulanic acid, cephalosporins, carbapenems, vancomycin and linezolid.^2^ |
| 3. Appropriate empiric antibiotic therapy: antibiotic agent or combination with *in vitro* activity against the *S. aureus* isolate that was commenced at the onset of the BSI, before the antibiogram of the isolate was known.^3^ |
| 4. Central venous catheter (CVC): an indwelling venous catheter that was inserted into the central venous system with the catheter tip positioned within the superior/inferior vena cava or right atrium, such as Hickman, Port-A-Cath, or central venous pressure (CVP) catheters.^3^ |
| 5. Chronic lung disease: childhood condition that primarily involves the alveoli and perialveolar tissues, leading to derangement of gas exchange and diffuse infiltrates on radiographs which include interstitial lung diseases and specific conditions such as cystic fibrosis and bronchiectasis.^4^ |
| 6. Coagulopathy: A prothrombin time of ≥2 seconds, an activated partial thromboplastin time of ≥60 seconds or a fibrinogen level of <2 μmol/L.^5^ |
| 7. Community-acquired MSSA or MRSA: *S. aureus* infection present on admission confirmed on a blood culture specimen obtained on the day of admission to RCWMCH or referring facility (calendar day 1), 2 days before admission or the calendar day after admission (calendar day 2).^6^ |
| 8. Co-morbidity – any underlying chronic disease the patient had at the time of *S. aureus* BSI diagnosis |
| 9. Date of onset of *S. aureus* BSI: The date on which the first positive blood culture for *S. aureus* was performed.^3^ |
| 10. Definitive antimicrobial therapy: antimicrobial therapy administered to the patient after confirming the *S. aureus* BSI and susceptibility from blood culture results ^3^ |
| 11. Effective antibiotic therapy: administration of an antibiotic either empirically or definitively to which the *S. aureus* isolate was susceptible |
| 12. Elevated C-reactive protein (CRP) concentration: any CRP reading above the NHLS reference range of 0-10 mg/L |
| 13. Elevated procalcitonin (PCT) concentration: any PCT value above 0.5 μg/L |
| 14. Empiric antimicrobial therapy: antimicrobial treatment administered to the patient prior to obtaining a definitive diagnosis for the *S. aureus* BSI from blood culture results |
| 15. Fever: an axillary temperature greater or equal to 38 degrees Celsius.^3^ |
| 16. Hepatic dysfunction: a ≥2-fold increase of serum aspartate aminotransferase and/or serum alanine aminotransferase concentration and/or a total bilirubin in a child more than 28 days old of >70 μmol/L.^7^ |
| 17. Healthcare-associated MSSA or MRSA: *S. aureus* infection confirmed on a blood culture specimen obtained on or after the 3^rd^ calendar day of admission.^6^ |
| 18. HIV status: (1) HIV-infection in a child <18 months of age: a positive HIV DNA PCR result confirmed by either a quantitative HIV RNA PCR or repeat HIV DNA PCR on a separate sample, or in a child ≥18 months of age: 2 positive serological test results (HIV ELISA or HIV rapid test) or a positive HIV DNA PCR result confirmed by either a quantitative HIV RNA PCR or repeat HIV DNA PCR test, (2) HIV-uninfected child: a child with a negative HIV serological test (HIV ELISA or HIV rapid test) or a negative virological test for HIV (e.g. HIV DNA PCR). (3) Unknown HIV status: a child with no history of HIV testing, no record of HIV testing in the NHLS laboratory database and whose mother’s HIV status prior to discharge from RCWMCH was unknown.^3^ |
| 19. Immunosuppressive therapy: Current treatment or treatment 2 weeks prior to date of onset of BSI with corticosteroids for more than one week or treatment with any immune modulating agent such as used in transplant patients.^8^ |
| 20. Leukocytosis: an absolute white cell count above the normal reference range for age, according to the NHLS reference values |
| 21. Moderate and severe underweight: weight-for-age Z-score (WFAZ) between -2 and -3 standard deviations (SD) and a WFAZ <-3 SD below the median WHO growth reference standards, respectively.^9^ |
| 22. MRSA: *S. aureus* isolate that is resistant to cefoxitin/oxacillin on AST.^6^ |
| 23. MSSA: *S. aureus* isolate that is susceptible to cefoxitin/oxacillin on AST.^10^ |
| 24. Neutropaenia: an absolute neutrophil count less than 1500 cells/μL; severe neutropenia: an absolute neutrophil count of less than 500 cells/μL |
| 25. Renal dysfunction: a serum creatinine concentration above the normal age-related range.^11^ |
| 26. Respiratory failure: the need for mechanical ventilatory support.^3^ |
| 27. *S. aureus* bloodstream infection: any case/patient whose blood culture specimen was positive for *S. aureus.*^12^ |
| 28. Shock: the presence of any one of the following criteria – hypotension for age; or any two of the following signs of inadequate tissue perfusion such as prolonged capillary refill, oliguria, metabolic acidosis or elevated tissue lactate for which patient required ionotropic support.^13^ |
| 29. Site of infection: the clinical site of infection as determined by the attending clinician |
| 30. Thrombocytopaenia: a platelet count less than 150x10^9^/L |
| 31. Underweight: weight-for-age Z-score < -2 |

**References used for study definitions**

1. World Health Organization. The global prevalence of anaemia in 2011. World Health Organization. 2015. [https://apps.who.int/iris/handle/10665/177094, accessed on 20 June 2023](https://apps.who.int/iris/handle/10665/177094,%20accessed%20on%2020%20June%202023%20)

2. Magiorakos AP, Srinivasan A, Carey RB, Carmeli Y, Falagas ME, Giske CG, et al. Multidrug-resistant, extensively drug-resistant and pandrug-resistant bacteria: an international expert proposal for interim standard definitions for acquired resistance. Clin Microbiol Infect. 2012; 18(3):268-81. PubMed PMID: 21793988.

3. Dame JA, Beylis N, Nuttall J and Eley B. Pseudomonas aeruginosa bloodstream infection at a tertiary referral hospital for children. BMC Infectious Diseases. 2020; 20:729; doi.org/10.1186/s12879-020-05437-1

4. Nevel RJ and Sharma GD. Childhood Interstitial Lung Disease (ChILD). Emedicine 2020. <https://emedicine.medscape.com/article/1003631-overview> accessed on 20/08/2022

5. Goldstein B, Giroir B, Randolph A. International pediatric sepsis consensus conference: definitions for sepsis and organ dysfunction in pediatrics. Pediatric critical care medicine. 2005; 6(1):2-8.

6. National Institute for Communicable Diseases. Methicillin-Resistant *Staphylococcus Aureus* (MRSA). <https://www.nicd.ac.za/diseases-a-z-index/methicillin-resistant-staphylococcus-aureus-mrsa/> accessed on 05/06/2022

7. Dellinger RP, Levy MM, Rhodes A, Annane D, Gerlach H, Opal S.M, et al. Surviving Sepsis Campaign: international guidelines for management of severe sepsis and septic shock. Intensive care medicine. 2013; 39(2):165-22

8. [Gutierrez-Dalmau](https://link-springer-com.ezproxy.uct.ac.za/article/10.2165/00003495-200767080-00006#auth-Alex-Gutierrez_Dalmau) A [and Campistol](https://link-springer-com.ezproxy.uct.ac.za/article/10.2165/00003495-200767080-00006#auth-Josep_M_-Campistol) JM. Immunosuppressive Therapy and Malignancy in Organ Transplant Recipients. A Systematic Review. [Drugs](https://link-springer-com.ezproxy.uct.ac.za/journal/40265), 2007;67;1167–1198

9. World Health Organisation. Handbook IMCI: Integrated Management of Childhood illnesses. 2005; ISBN 92 4 154644 1

10. National Institute for Communicable Diseases. *Staphylococcus Aureus* <https://www.nicd.ac.za/diseases-a-z-index/staphylococcus-aureus/> accessed on 05/06/2022

11. Boer DP, de Rijke YB, Hop WC, Cransberg K, Dorresteijn EM. Reference values for serum creatinine in children younger than 1 year of age. Pediatric nephrology. 2010; 25(10):2107-13

12. McMullan BJ, Campbell AJ, Blyth CB, McNeil C, Montgomery PC, Tong SYC et al. Clinical Management of *Staphylococcus aureus* Bacteremia in Neonates, Children and Adolescents. Pediatrics. 2020; 146; 3: e20200134; [doi.org/10.1542/peds.2020-0134](https://doi.org/10.1542/peds.2020-0134)

13. Dellinger RP, Levy MM, Rhodes A, Annane D, Gerlach H, Opal S.M, et al. Surviving Sepsis Campaign: international guidelines for management of severe sepsis and septic shock. Intensive care medicine. 2013; 39(2):165-22

**Supplementary table 2.** Episodes and incidence risk of *Staphylococcus aureus* bloodstream infections/1000 admissions per year during 2018-2022

| Year | Total Admissions | MSSA | MRSA | Total episodes |
| --- | --- | --- | --- | --- |
| 2018 | 20,792 | 51 (2.5) | 7 (0.3) | 58 (2.8) |
| 2019 | 20,070 | 40 (2.0) | 11 (0.6) | 51 (2.5) |
| 2020 | 15,161 | 41 (2.7) | 3 (0.2) | 44 (2.9) |
| 2021 | 17,064 | 53 (3.1) | 2 (0.1) | 55 (3.2) |
| 2022 | 18,647 | 35 (1.9) | 2 (0.1) | 37 (2.0) |
| Total | 91,734 | 220 (2.4) | 25 (0.3) | 245 (2.7) |

MSSA, methicillin-susceptible *Staphylococcus aureus*; MRSA, methicillin-resistant *Staphylococcus aureus*

**Supplementary table 3**. Associated comorbidities among participants at the time of *Staphylococcus aureus* bloodstream infection diagnosis

| MSSA (N=216) | | MRSA (N=24) | |
| --- | --- | --- | --- |
| Comorbidity | **Number (%)** | **Comorbidity** | **Number (%)** |
| None | 125 (58) | None | 3 (12.5) |
| Malignancy | 13 (6) | Malignancy | 1 (4.2) |
| Cardiac disease (congenital) | 21 (9.7) | Cardiac disease (congenital) | 8 (33) |
| Burns | 13 (6) | Burns | 7 (29) |
| Renal diseases | 4 (1.9) | Renal diseases | 1 (4.2) |
| Chronic liver disease | 3 (1.4) | Chronic liver disease | 0 (0) |
| Chronic lung disease | 3 (1.4) | Chronic lung disease | 0 (0) |
| HIV infection | 5 (2.3) | HIV infection | 0 (0) |
| Others (cystic fibrosis, primary immunodeficiency, sickle cell disease, severe acute malnutrition, hydrocephalus, aplastic anaemia) | 29 (13.4) | Others (cystic fibrosis, severe acute malnutrition, hydrocephalus, aplastic anaemia) | 4 (1.7) |

MSSA, methicillin-susceptible *Staphylococcus aureus*; MRSA, methicillin-resistant *Staphylococcus aureus*; HIV, Human immunodeficiency virus

**Supplementary table 4.** Comparing results with a previous study on *Staphylococcus aureus* bloodstream infections at Red Cross War Memorial Children’s Hospital (2007-2011/2018-2022)^4^

|  | 2018-2022 | 2007-2011 | p-value |
| --- | --- | --- | --- |
| Total number of BSIs | 240 | 365 |  |
| Male: Female, number (%) | 146 (60.8): 94(39.2) | 193 (53): 172 (47) | 0.05 |
| Weight-for-age z-score <-2, n/N (%) | 70/240 (29.2) | 131/331 (39.6) | 0.01 |
| HIV status, n/N (%) |  |  | <0.001 |
| Infected | 5/240 (2.1) | 51/258 (19.8) |  |
| Exposed uninfected | 50/240 (20.8) | 48/258 (18.6) |  |
| Unexposed uninfected | 142/240 (59.2) | 50/258 (19.4) |  |
| Unknown | 43/240 (17.9) | 109/258 (42.2) |  |
| *S. aureus* BSI classification, n/N (%) |  |  | 0.9 |
| Community-acquired  infection | 160/240 (66.7) | 239/357 (66.9) |  |
| Healthcare-associated  infection | 80/240 (33.3) | 118/357 (33.1) |  |
| MSSA: MRSA, n/N (%) | 216 (90): 24 (10) | 270 (74.0): 95(26.0) | <0.001 |
| Community-acquired MRSA, n/N (%) | 5/160 (3.1) | 6/239 (2.5) | 0.7 |
| Healthcare-associated MRSA, n/N (%) | 19/80 (23.8) | 69/118 (58.5) | <0.001 |
| Frequent primary diagnoses, n/N (%) |  |  | 0.01 |
| BSI without focus | 73/240 (30.4) | 110/337 (32.6) |  |
| Skin and soft tissue infection | 60/240 (25) | 58/337 (17.2) |  |
| Bone and joint infection | 33/240 (13.8) | 39/337 (11.6) |  |
| Pneumonia | 24/240 (10) | 73/337 (21.7) |  |
|  |  |  |  |
| 28-day mortality, n/N (%) | 20/240 (8.3) | 32/365 (8.8) | 0.9 |

MSSA, methicillin-susceptible *Staphylococcus aureus*; MRSA, methicillin-resistant *Staphylococcus aureus;* BSIs, bloodstream infections; HIV, Human immunodeficiency virus
